# In Vitro Effects of Postmetabolites from *Limosilactobacillus fermentum* 53 on the Survival and Proliferation of HT-29 Cells

**DOI:** 10.3390/microorganisms12071365

**Published:** 2024-07-03

**Authors:** Veselina Moskova-Doumanova, Anita Vaseva, Ralitsa Veleva, Kirilka Mladenova, Denitsa Melniska, Jordan Doumanov, Pavel Videv, Tanya Topouzova-Hristova, Lili Dobreva, Nikoleta Atanasova, Svetla Danova

**Affiliations:** 1 Faculty of Biology, Department of Cell and Developmental Biology, Sofia University St. Kliment Ohridski, 8 Dragan Tsankov Blvd., 1164 Sofia, Bulgaria; moskova@biofac.uni-sofia.bg (V.M.-D.); topouzova@biofac.uni-sofia.bg (T.T.-H.); 2 Faculty of Biology, Department of Biochemistry, Sofia University St. Kliment Ohridski, 8 Dragan Tsankov Blvd., 1164 Sofia, Bulgaria; k_mladenova@biofac.uni-sofia.bg (K.M.); doumanov@biofac.uni-sofia.bg (J.D.);; 3 Bulgarian Academy of Sciences, Stefan Angeloff Institute of Microbiology, 26, Acad. Georgi Bonchev Street, 1113 Sofia, Bulgaria; lili.ivailova1@gmail.com (L.D.); nikoletaatanasova21@gmail.com (N.A.)

**Keywords:** *Limosilactobacillus fermentum*, TEER, postmetabolites, HT-29 cells

## Abstract

Naturally fermented dairy products are an important component of the human diet. They are a valuable source of nutrients as well as vitamins and minerals. Their importance as a source of probiotic bacterial strains should not be overlooked. A number of studies highlight the positive effects of species of the probiotic lactic acid bacteria on the intestinal microbiome and the overall homeostasis of the body, as well as a complementary treatment for some diseases. However, data on the effects on the intestinal epithelial cells of postmetabolites released by probiotic bacteria are incomplete. This is likely due to the fact that these effects are species- and strain-specific. In the present study, we investigated the effects of postmetabolites produced by a pre-selected candidate probiotic strain *Limosilactobacillus fermentum* on HT-29 intestinal epithelial cells. Our data showed a pronounced proliferative effect, evaluated by flow cytometry, quantification of the cell population and determination of the mitotic index. This was accompanied by the stabilization of the cell monolayer, measured by an increase in TEER (transepithelial electric resistance) and the reorganization of actin filaments. The data obtained are a clear indication of the positive effects that the products secreted by *L. fermentum* strain 53 have on intestinal epithelial cells.

## 1. Introduction

Probiotic strains of bacteria have been the subject of active research in recent decades, although fermented foods, which are their main source, such as yogurt, various types of cheese, katuk, kefir, boza, sauerkraut, Korean kimchi, kombucha and many others, have been used by humanity since ancient times. The first suggestion of the health benefits of probiotics dates back to the early 20th century, when Elia Metchnikoff attributed the long life of Bulgarian peasants to their consumption of fermented milk containing lactic acid bacteria (LAB) [1]. Most of the bacteria with probiotic potential are from the genera *Lactobacillus*, *Bifidobacteria*, *Streptococcus*, *Propionibacterium*, *Leuconostoc* and *Enterococcus*; however, the characteristics and probiotic effects are species- and even strain-specific [1].

The main reason for modern humans to take probiotics is for the restoration of the normal gut microbiota after antibiotic treatment. In fact, probiotics have a wide range of applications, as they have a proven effect against severe, difficult-to-treat diseases such as cancer [2,3], diabetes [4,5] and obesity [6,7,8], as well as involvement in the metabolism of lipids and carbohydrates [9], and can even affect the central nervous system and brain activity [10].

Probiotic bacteria (pro-for and bio-life) exert their positive effects through the postbiotics they release. “Postbiotics” is defined as “preparation of inanimate microorganisms and/or their components that confers a health benefit on the host”, i.e., dead microorganisms or the remains of them after lysis” [11]. Also highly disused are bacterial metabolites or byproducts of bacterial metabolism, which do not contain live bacteria and are non-toxic and pathogenic. In the present work, they are pointed as postmetabolites. During fermentation, mainly bacteriocins, peptides, organic acids, acetaldehydes, alcohols, hydrogen peroxide and others were reported to be produced, all of which have enormous potential in fighting pathogenic microorganisms and also in supporting epithelial cells in the body [10]. Postbiotics can be secreted by living bacteria or released into the medium after their lysis. They most often affect the body by binding to Toll-like signal transduction receptors (TLRs) located on the surface of intestinal epithelial, dendritic and other cells associated with the immune response [11].

Much of the research on probiotics and postbiotics has focused on their action on the morphology of healthy enterocytes and influence on various pathological conditions of these cells. Some bacteria of the normal gut microbiota have been found to contain mitogens that stimulate DNA synthesis and epithelial cell proliferation [3]. The gut microbiota strengthens the intestinal epithelium and improves its barrier functions [12,13,14] by stabilizing tight junctions [15], changes in the cytoskeleton and redistribution of its proteins [16,17], while others cause disorders and inflammatory processes in these cells. For this, it is important to determine the exact mode of action of individual strains and select those that directly inhibit pathogenic microorganisms, or those that enrich and support the “good” bacteria in the natural microbiota [18], or possess positive effects on epithelial cells.

The aim of the present study was to determine the postbiotic effects of pre-selected candidate probiotic strain *Limosilactobacillus fermentum* 53 (*Lf53*) on the key characteristics of the HT-29 intestinal epithelial cell line. Our hypothesis is that the Lf 53 strain may possess a pronounced positive effect on intestinal epithelial cells under normal conditions. This will make it a suitable candidate for application as a probiotic because it can support the healing of the intestinal mucosa in case of daily damage from food components. Therefore, we estimated the proliferative effects, viability and mitotic effects produced during the fermentation of spent cultures, postmetabolites and their role and mechanism of stabilization of the epithelial cell monolayer.

## 2. Materials and Methods

### 2.1. Microorganisms, Culture Conditions and Preparation of Spent LAB Cultures (Postmetabolites)

*Limosilactobacillus fermentum strain* 53 is a part of the laboratory collection of the “Stephan Angeloff” Institute of microbiology, Bulgarian Academy of Sciences. The strain was characterized as a candidate probiotic with a broad spectrum of antagonistic activity [19]. The strain was stored at −80 °C in MRS broth (HiMedia, Mumbai, India), supplemented with 20% *v*/*v* glycerol. Prior to the assay, *L. fermentum* 53 was cultured twice in MRS broth for 24 h. The postmetabolites were obtained during the 24 h fermentation at 37 °C in MRS broth inoculated with 10% *v*/*v* from overnight culture (in late exponential CFSs). The cells were harvested after centrifugation for 10 min at 6000 rpm (centrifuge Hermle, Germany) and the cell-free supernatants (CFS/postmetabolites) were collected. They were filtered with syringe filters (Millipore, Darmstadt, Germany, 0.22 µm) and stored at −20 °C. To simulate in vivo conditions, the CFSs were not neutralized. The protein content of postmetabolites from spent cultures in MRS broth and in pure MRS broth was determined by the BCA Bradford method, as described by Smith et al., 1985 [20].

### 2.2. Cell Cultures

Human epithelial-like adenocarcinoma HT-29 cell line (ATCC^®^ No. HTB-38™) was kindly provided by prof. Ivan Iliev, Bulgarian Academy of Sciences. Cells were grown in 25 cm^2^ ‘CELLSTAR^®^’ flasks (Corning, New York, NY, USA), at standard conditions in humidified atmosphere with 5% CO_2_, at 37 °C, in Dulbecco‘s modified eagle medium (DMEM, Sigma–Aldrich, St. Louis, MO, USA), supplemented with 10% fetal bovine serum (FBS, Sigma–Aldrich) and 1% (*v*/*v*) antibiotic–antimycotic solution (penicillin 100 U/mL, streptomycin 100 μg/mL and amphotericin B 0.25 μg/mL, Sigma–Aldrich).

Each of the experiments was performed in three variants:

1. Untreated cells, maintained in DMEM, serving as a control;
2. An experimental group of cells cultured in the presence of *L. fermentum* 53 postmetabolites in MRS broth (spent LAB cultures in MRS broth/CFSs), dissolved at different concentrations in DMEM;
3. Cells cultured in the presence of pure MRS broth, dissolved at different concentrations in DMEM, in order to differentiate the effects of the products released by the bacteria from those of the MRS broth components.

### 2.3. Cell Viability Tests (Crystal Violet Staining)

For the determination of cellular viability and morphology, cells were seeded in 96-well plates (CELLSTAR^®^, Greiner Bio-One, Kremsmünster, Austria) 24 h before treatment, at an initial concentration of 1 × 10^4^ cells/well. Cells were then treated with collected bacterial postmetabolites or MRS broth, dissolved in DMEM, with a protein content concentration ranging from 0.5 mg/mL to 2 mg/mL. The proportion of viable cells was determined by crystal violet staining. At 24 h post-treatment, cell monolayers were washed with phosphate-buffered saline (PBS) and fixed with 4% formaldehyde in PBS for 20 min. The plates were washed with distilled water and 1% crystal violet solution was added to each well for 20 min at room temperature. After washing, pictures of cell morphology were taken using an inverted microscope Leica DMi1 supplied with Leica MC170 camera. Cells were then water-washed, air-dried, and the protein-bound dye (which corresponds to the number of cells) was solubilized with 10% acetic acid. The optical density of each sample was measured spectrophotometrically at λ = 570 nm, using an Epoch Microplate spectrophotometer with Gen5 Data Analysis software. Values were calculated as a percentage of control cells (incubated under the same conditions in DMEM, without MRS or CFSs).

### 2.4. Mitotic Index Determination 

Cells were subcultured in sterile 6-well Costar^®^ plates (Corning, New York, NY, USA) at a concentration of 5.10^4^ cells/mL on pre-sterilized coverslips. In each of the plates, cells from one well served as a control (untreated cells, maintained in DMEM), in two of the wells, the cells were treated with pure MRS dissolved in DMEM (at protein concentrations of 0.5 mg/mL and 1 mg/mL) and in two others, MRS containing bacterial postmetabolites/CFSs (dissolved in DMEM, at concentrations of 0.5 mg/mL and 1 mg/mL protein) was added to the cells. Every 24 h for 6 days, slides from each well in the corresponding plates were fixed with 70% ethanol for 20 min, then stained with Giemsa solution for 5 min, washed repeatedly with dH_2_O, air-dried for at least 24 h and mounted on glass slides by Gel Mount^TM^ (Sigma, St. Louis, MO, USA). Determination of the mitotic index was performed by counting at least 1000 cells per slide, at randomly selected locations, using an inverted microscope (Leica DMi1). The mitotic index (MI) was calculated according to the formula:

MI = NM/NT × 100,

where

NM is the number of cells in mitosis and

NT is the total number of cells counted.

The results obtained are presented in percentages [21].

### 2.5. Assessment of Transepithelial Electrical Resistance (TEER)

The HT-29 cells were subcultured in sterile 6-well Corning^®^ Transwell^®^ plates (USA) at a concentration of 1.10^5^ cells/mL. Post-bacterial metabolites or MRS broth were added only to the upper compartment of the well, i.e., from the apical part of the cells in a concentration of 0.5 mg/mL protein content. In this way, an attempt was made to simulate the conditions in vivo, in which the gut microbiota, and their postmetabolites respectively, are in the lumen of the intestine, from the apical part of the cells. Every 24 h, immediately before the measurement, the culture medium was replaced with the corresponding one. A transepithelial resistance voltmeter EVOM2 (World Precision Instruments, Inc., Sarasota, FL, USA) was used to measure cell monolayer resistance. One electrode was placed over the semipermeable membrane with the monolayer of cells and the other in the well with the medium. The measurement was carried out every 24 h for 10 days. TEER values measured on the first day of cultivation were taken as 0, and values measured on subsequent days were recalculated relative to it.

### 2.6. FACS Analysis of Cell Cycle

Possible changes in cell cycle phases induced by bacterial postmetabolites were investigated by FACS analysis. Cells were cultured in 6-well sterile Costar^®^ plates (Corning, USA) at an initial concentration of 5.10^4^ cells/mL, under standard conditions for 24 h before treatment. Two of the wells were then treated with MRS broth in DMEM (0.5 mg/mL protein content), another two with MRS containing postmetabolites from *L. fermentum* 53 (0.5 mg/mL) and the remaining two wells served as control 48 h after treatment; one well of each variant was incubated for 4 h with colcemid (0.1 μg/mL) to block the cell cycle. Cell labeling was performed with Guava^®^ Cell Cycle Reagent (CYTEKBioscience, Fremont, CA, USA) according to the manufacturer’s instructions, namely, after the incubation period, the cells were centrifuged for 5 min at 450 g to remove the culture medium and washed with sterile PBS, by centrifugation for 5 min at 450 g. After removing the supernatant, the cells in the remaining PBS were dropped into ice-cold 70% ethanol while vortexing for fixation. Fixed cells were stained with cell cycle reagent (Guava^®^ Cell Cycle Reagent). A Guava^®^ easyCyte™ Flow Cytometer (Luminex, Belgium) was used for reading.

### 2.7. Actin Filaments Visualization

Twenty-four hours before treatment, HT-29 cells were seeded in 6-well Costar^®^ plates (Corning) on sterile coverslips, at two different starting concentrations −5.10^4^ cells/mL and 1.10^5^ cells/mL. This was followed by treatment with pure MRS dissolved in DMEM or MRS with Lf 53 postbiotics at a concentration of 0.5 mg/mL. In the control wells, the medium was replaced with a fresh one. After 48 h treatment, the medium from all wells was removed, the cells were washed with PBS (pH 7.4), permeabilized by 0.5% Triton X-100 for 5 min, washed with 1 × PBS and blocked with 1% BSA for 30 min. For staining, cells were incubated with 100 nM TRITC (tetramethylrhodamineisothiocyanate)-phalloidin (Sigma-Aldrich) for 1 h in the dark at room temperature, washed with PBS, then cell nuclei were stained with 100 nM DAPI (DeltaVision Ultra™, GE Healthcare, Chicago, IL, USA) for 5 min, again in the dark. Both dyes were dissolved in PBS. After staining, the slides were washed with dH_2_O and mounted on glass slides using Gel Mount^TM^ (Sigma, St. Louis, MA, USA). Cells were visualized using a fluorescence microscope (Nikon TiU, Tokyo, Japan).

### 2.8. Statistical Analysis

All experiments were performed in triplicates. Results reported in the text are expressed as means and standard error (±SE). Statistical analysis was performed by one-way Anova using Origin Pro 9 software; *p* = 0.05 was accepted as a statistically significant difference.

## 3. Results

### 3.1. Positive Effect of L. fermentum 53 Postmetabolites on the Growth Characteristics of HT-29 Cells

**Figure 1:**
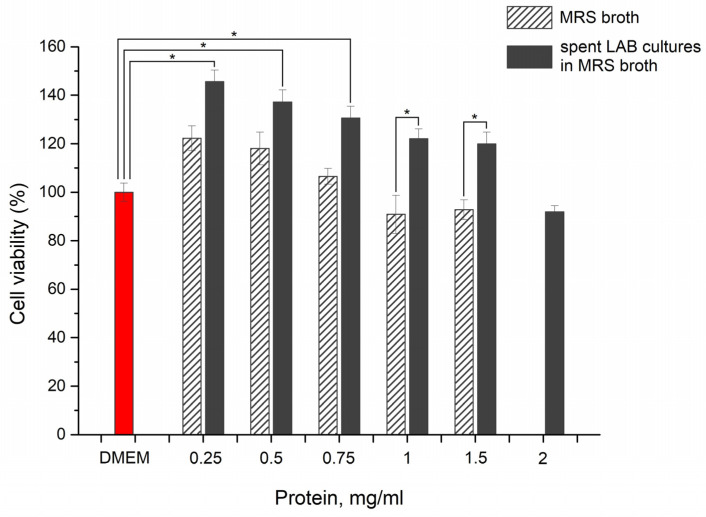
Assessment of cell viability. Evaluation of the effect of postmetabolites released from *L. fermentum* (Lf 53) and MRS broth on the growth characteristics of HT-29 cells. Amount of cells cultured in pure DMEM medium (control cells) was considered as 100%; *p* = 0.05 (*).

A model microplate system with a subconfluent monolayer of eukaryotic cells was used to evaluate whether microbial postmetabolites have an effect on HT-29 cell growth. The cells were treated with different concentrations of postmetabolites released during the LAB fermentation in MRS broth and added to DMEM medium. In order to repeat the experimental conditions and adequately evaluate the results, dilutions were made relative to the protein content of MRS or of MRS with postmetabolites (CFSs). Cristal violet staining clearly indicated that postmetabolites released in the culture medium have a positive influence on the survival of HT-29 cells (Figure 1). At all dilutions tested, the cell amount was greater than that of the control cells cultured in pure DMEM medium. This effect was in a concentration-dependent manner, and most noticeable at low treatment concentrations, and as the concentration of bacterial postmetabolites increased, the positive effect decreased. At the concentrations we tested, the cell proportion ranged from 148% (for 0.25 mg/mL) to 120% (for 1.5 mg/mL) compared to the control. The data were statistically significant for the protein content concentration from the LAB-spent MRS in DMEM from 0.25 mg/mL to 0.75 mg/mL. Only at the highest concentration tested, 2 mg/mL protein content, was the amount of cells very slightly reduced as compared to the control (93%), but these data were not statistically significant.

To ensure that the observed positive effect was due solely to the released bacterial postmetabolites and not the MRS broth components, the effect of the latter on the survival of HT-29 cells was also tested. We found a dose-dependent positive effect that decreased with the increase in the tested concentrations, but the data were statistically significant only at a 1 mg/mL and 1.5 mg/mL protein content. Therefore, we believe that the observed positive effect on cell viability is at least due to substances released by *L. fermentum* 53 into the culture medium. The increase in the cell viability signal could be due to the increase in the cell number or enlargement of cells, accompanied by the stimulation of protein synthesis.

### 3.2. Transepithelial Electrical Resistance (TEER)

**Figure 2:**
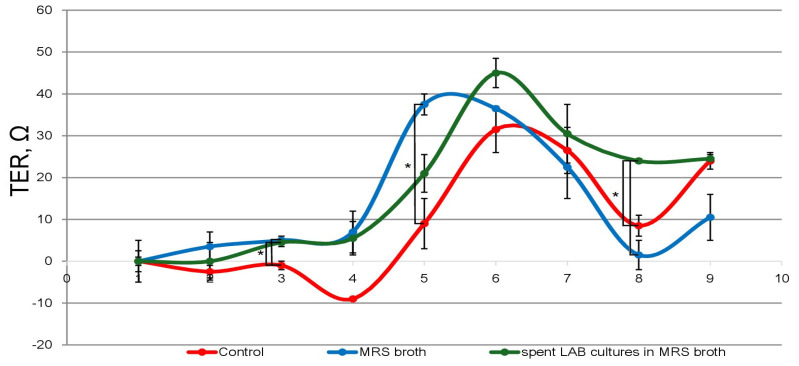
Changes in the transepithelial electrical resistance (TEER) of HT-29 cells’ monolayer for the period of 9 days. Red line−control cells grown in DMEM (no MRS, or LAB-spent MRS added), blue−cells grown in DMEM, supplemented with sterile MRS broth (0.5 mg/mL protein content), grey−cells grown in DMEM, supplemented with CFS from LAB cultures in MRS broth (0.5 mg/mL protein content) *p* = 0.05 (*).

Determination of changes in transepithelial resistance is a convenient and informative method providing information on cell layer integrity and paracellular ion transport [22,23]. Changes in the closure rate and stability of the HT-29 cell monolayer were monitored by changes in the transepithelial electrical resistance (Figure 2). During the first three days, low, constant values of transepithelial resistance were observed in the control cells, maintained in DMEM, corresponding to the absence of a proper monolayer. A gradual increase in values followed, with a maximum on the sixth–seventh day and values of about 30 ohms, corresponding to the closing of the intercellular space and the formation of a proper and tight monolayer. In cells cultured in the presence of 0.5 mg/mL protein content, MRS broth formation of the dense monolayer was slower, with low, constant TEER values observed during the first four days. These were followed on day 5–6 by a sharp increase in values above those of the control cells, up to 36–37 ohms. This peak, in addition to being characterized by higher values, preceded by about 24 h the peak observed in the control cells.

In cells cultured in the presence of bacterial postmetabolites, the change in transepithelial resistance by day 4 followed that of cells cultured in DMEM supplemented with sterile MRS broth. This was followed by a sharp rise in values within 48 h. The TEER peak was measured on day 6. The values of 45 ohms exceeded those measured in the control cells and cells cultured in the presence of MRS broth. After this peak, there was a reduction in values at day 7–9 of cultivation to 25–30 ohms; however, they still remained higher than the maximum values determined for the other variants of the experiment, which is an indication of the stabilization of the monolayer.

These data are indicative of a stabilizing effect of the postmetabolites released from *L. fermentum* 53 on the cell monolayer. The same TEER values measured for the monolayer of cells cultured in DMEM and in the presence of bacterial post-metabolites on day 9 we consider to be suggestive of the latter not adversely affecting the membrane permeability, paracellular ion transport and barrier function of epithelial cells. Probably, components produced by the bacterial cells and secreted in the culture medium lead to the formation of a more resistant layer of enterocytes, exhibiting better barrier functions. As can be seen from Figure 2, the dynamics of the TEER values for the control cells differ from those of cells cultured in the presence of bacterial postmetabolites. The permanently higher values for the latter are evident, which we perceive as an indication of a much faster closure of the monolayer. We believe that this is the positive effect of postmetabolites, because daily, the intestinal epithelium suffers mechanical damage from harder food particles, and the rapid repair of such injuries can be critical.

### 3.3. In Vitro Effects of L. fermentum Postmetabolites on Cellular Morphology and Organization of Actin Cytoskeleton of HT-29 Cells

**Figure 3:**
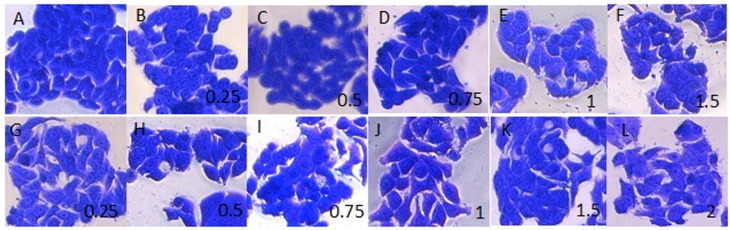
Effect of *L. fermentum* Lf53 postmetabolites on cellular morphology. (**A**)—control, (**B**–**F**)—cells cultivated in the presence of MRS broth, (**G**–**L**)—cells cultivated in the presence of spent LAB cultures in MRS broth (protein amount, in mg/mL is indicated on pictures). Magnification 20×.

To estimate whether the observed positive effects of postbiotics on cell viability and TEER were due to changes in cell morphology and organization, we performed microscopic analysis of the monolayer and actin cytoskeleton. We did not observe significant changes in the cell morphology after treatment with both pure MRS broth and medium containing bacterial postmetabolites/CFSs (Figure 3). Cells from the HT-29 line have an epithelium-like morphology, growing as clusters of cells in which different contacts are established between the cells (https://www.atcc.org/products/htb-38, accessed on 27 March 2024). These morphological features were preserved in cells grown in the presence of MRS broth and MRS broth with bacterial postmetabolites at a concentration of 0.5 mg/mL and 1 mg/mL protein content. Consistent with the data for a positive effect of low concentrations of postmetabolites on cell viability, we also observed more clusters containing higher numbers of cells when cells were treated with 0.5 mg/mL CFSs.

**Figure 4:**
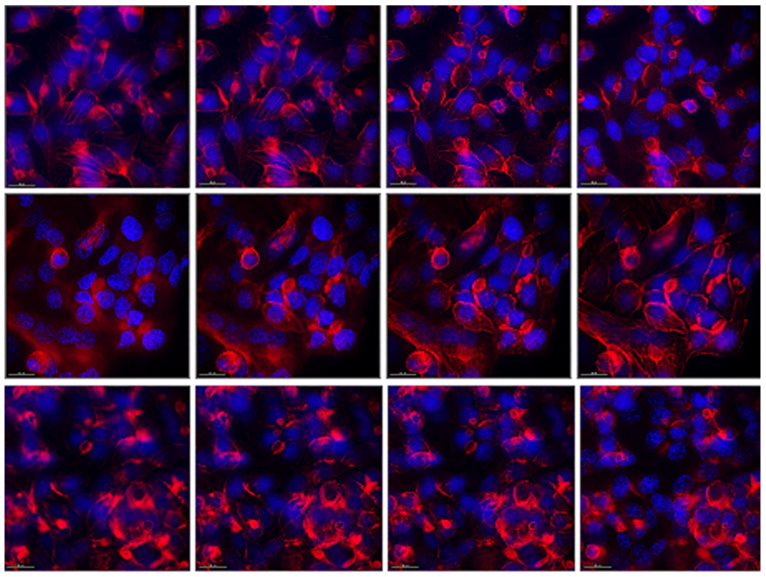
Fluorescent staining of actin filaments in HT-29 cells. Upper row—control cells maintained in DMEM; middle row—cells cultured for 48 h in the presence of MRS, 0.5 mg/mL protein content in DMEM, lower row—cells cultured for 48 h in the presence of CFS 0.5 mg/mL protein content. Left to right—bottom to top of the cells. Magnification 40×.

In the control cells, we observed actin filaments organized in the form of clearly visible stress fibrils ending at the cell periphery. Around the nuclei of the cells, the actin network was looser, and thickenings were observed in places at the periphery, probably corresponding to cell-to-cell contacts (Figure 4, upper row). Bacterial postmetabolites visibly affected the distribution of actin filaments (Figure 4, lower row). Much fewer stress fibrils were observed, and the actin filaments were much more compacted, mainly located under the cellular membrane. This indicates a much greater number of cell-to-cell contacts and cellular polarization. This is in accordance with the observations obtained from the previous experiments, namely that the bacterial postmetabolites from L. fermentum succeed to a large extent in stabilizing the monolayer of intestinal epithelial cells and improving their barrier function. In cells cultured in the presence of pure MRS medium, a slight effect on the actin cytoskeleton was observed (Figure 4, middle row). The actin filaments were clearly visible, but more compacted. This indicates the presence of a greater number of cellular contacts and possible stabilization of the monolayer. No changes in the nucleus were observed in any of the studied groups.

### 3.4. Effects of L. fermentum Postmetabolites on Cell Cycle of HT-29 Cells

The effects of the bacterial postmetabolites on the actin cytoskeleton of HT-29 cells were minimal and were not sufficient to explain the monolayer stabilization detected by the TEER measurement. Therefore, we asked whether the observed effect as well as increased cellular viability were not due to changes in the growth characteristics of the cells, namely, changes in the rate of cell division and/or in the distribution of cells in the phases of the cell cycle.

**Figure 5:**
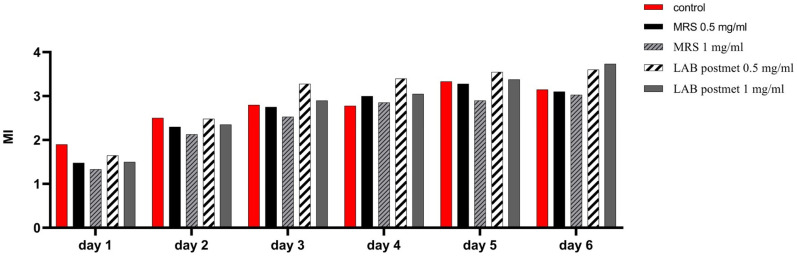
Changes in the mitotic index of HT-29 cells determined for the 6-day period. Control—cells cultured in DMEM without MRS or LAB postmetabolites added. Experimental groups—cells maintained for 6 days in DMEM, supplemented with MRS or LAB postmetabolites in DMEM (0.5 mg/mL or 1 mg/mL protein content).

We monitored for a period of 6 days after subculturing the changes in the mitotic index of cells cultured in the constant presence of bacterial postmetabolites (Figure 5). The effect of two concentrations determined relative to the protein content of the sample, namely 0.5 mg/mL and 1 mg/mL, was evaluated. By the 48th hour of cultivation, the highest mitotic index (MI) values were found for the control cells, while the values for the cells cultured in MRS broth and bacterial postmetabolites were lower than the control, but comparable to each other. This may be attributed to the adaptation of enterocytes to the unusual-for-them components added to the medium. From the 72nd hour of cultivation, throughout the period studied, the proportion of dividing cells when cultured in medium containing bacterial postmetabolites was higher compared to cells cultured in the presence of MRS broth as well as in standard cultural medium. We found that throughout the investigated period, the presence of MRS broth in the culture medium of eukaryotic cells had a slight inhibitory effect on cell division, which was expressed in a permanently lower MI, compared to the control cells. These data are a clear indication of the cell division-promoting effect exerted by bacterial postmetabolites released from *L. fermentum* 53. Once again, we observed a better effect at the lower treatment concentration of 0.5 mg/mL.

In an attempt to refine the mitosis-promoting action of bacterial postmetabolites, we followed their influence on the cell cycle of cultured cells by flow cytometry. We used only one concentration of 0.5 mg/mL, which in previous experiments showed a more pronounced effect.

**Figure 6:**
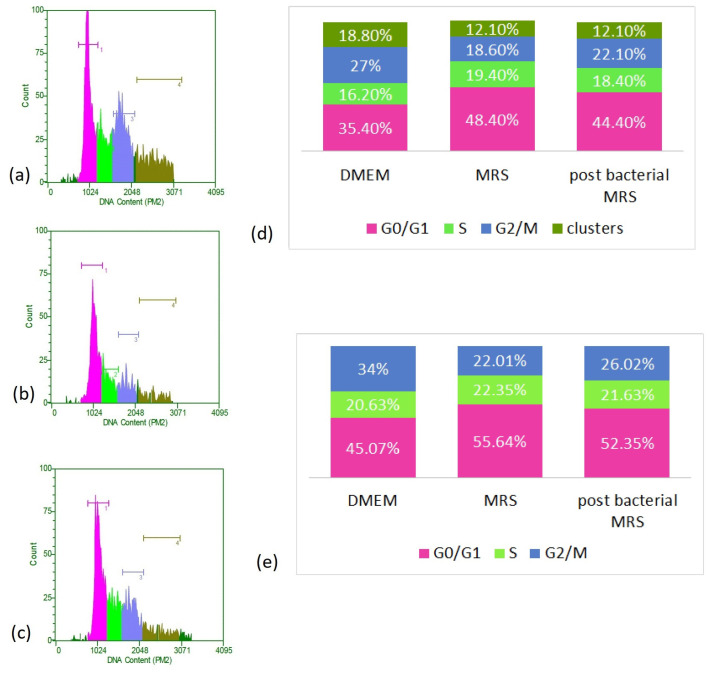
FACS analysis of cell cycle of HT-29 cells. (**a**) Control cells maintained in DMEM, (**b**) cells cultured for 48 h in DMEM supplemented with MRS, 0.5 mg/mL protein content, (**c**) cells cultured for 48 h in DMEM supplemented with CFS 0.5 mg/mL. (**d**) Distribution of cells into the different phases of the cell cycle, according to the DNA amount. (**e**) Redistribution of cells into the different phases of cell cycle, excluding polyploid cells and clusters.

We observed a clear redistribution of cells in the distinct stages of the cell cycle (Figure 6). We found that culturing the cells in the presence of MRS broth and bacterial postmetabolites led to a reduction in the proportion of polyploid cells and cell aggregates, decreasing from 18.8% in the control cells to 12.1% in both types of treated cells. In parallel, a decrease in tetraploid cells corresponding to the G2/M period was observed. This distribution is at the expense of an increase in the relative share of diploid cells that are at rest or have not yet activated the processes of replication and preparation for division. If the data are recalculated, disregarding polyploid cells and cell aggregates, it is found that culturing the cells in the presence of MRS broth and CFSs increases the proportion of diploid cells (located in G0/G1). Meanwhile, in the control cells, they constituted about 45% of the cell population, and their portion increased to over 50% under the remaining culture conditions (reaching 55.64% of cells grown in the presence of MRS broth and 52.35% of those cultured in the presence of bacterial postmetabolites). A slight increase, within about 1–1.5% of the proportion of cells in replication, was also observed as a result of the treatment compared to the controls. Given the fact that these are data obtained at the 48th hour of culturing, they support our observations obtained in the determination of the mitotic index, namely, that at this point in the culture, the proportion of dividing cells is highest in the control. The high percentage of cells cultured in the presence of bacterial postmetabolites that are in the S-period is also consistent with these data and correlates with the higher percentage of dividing cells found at a later stage. In addition, this is a likely indication of the presence of substances stimulating cell division (mitogens) released by *L. fermentum* 53.

## 4. Discussion

The positive effects of *lactobacilli* on the cells of the intestinal epithelium have been known for a while, but they are still the subject of intensive research due to the not fully understood mechanisms of their action. A challenge is also the fact that the postmetabolites they release, and correspondingly the effects they exert, are species- and in some cases strain-specific. In the present study, we aimed to follow the effects of postmetabolites released in the culture medium by the pre-selected candidate probiotic strain *Limosilactobacillus fermentum* 53 on the proliferative capacities and barrier functions of cultured human epithelial cells of the HT-29 line. We used this cell line as a model for the intestinal epithelium [24,25,26]. During cultivation, the cells retain their epithelium-like morphology, forming a well-organized monolayer. These characteristics were the main criterion for choosing this cell line as a model system in our study. Since one of the goals of our study was to evaluate the influence of bacterial postmetabolites precisely on the barrier functions of enterocytes (influence on cell proliferation and monolayer stability), we considered that this cell line was suitable, despite its cancerous origin. Our results showed a proliferation-stimulating effect of bacterial postmetabolites on intestinal epithelial cells. This effect was not unexpected, as evidence of the similar effects of other members of the genus *Lactobacillus* is available in the literature. Degradation of carbohydrates and proteins by fermentative bacteria has been shown to produce short-chain fatty acids that stimulate cell proliferation in the intestinal crypts [27]. However, the question still remains whether these mitogens are different for different strains of bacteria and how they affect DNA synthesis and the proliferative abilities of the cell. It was found that postbiotic p75 isolated from *Lactobacillus rhamnosus* GG regulates the expression of a number of genes involved in cell proliferation, development and apoptosis in HT 29 cells. It decreases the expression of pro-apoptotic and anti-proliferative genes and increases the expression of anti-apoptotic and pro-proliferative genes. It also activates Akt, which is part of the EGFR (epidermal growth factor receptor) signaling pathway. Thus, p75 contributes to cell proliferation by activating EGFR, by stimulating the genes for its ligands [28]. Another mechanism of influence on the proliferation of intestinal epithelial cells has been demonstrated for the strain *Lactobacillus reuteri* D8. It has been found to stimulate the proliferation of intestinal epithelial cells under normal conditions as well as participating in the repair of damaged epithelium under pathological conditions by increasing the expression levels of c-Myc, cyclins and Ki67 and participating in the activation of the Wnt/β-catenin pathway by increasing the expression of Wnt3, Lrp5 and β-catenin. These are one of the main molecules involved in both the stimulation of proliferation and the repair of damaged cells. This strain has also been shown to reduce the percentage of apoptotic cells and possibly contribute to decreased levels of TNF secretion [29].

Depending on the environmental conditions and the state of the cells, certain factors can stimulate both proliferation and cell quiescence or programmed cell death. Many of the key components leading to significant cell fate decisions are common to these signaling pathways, and the decision of subsequent events depends on the balance of signals [30]. In our experiments, the proportion of cells in the G1 period was significantly increased in the samples with the bacterial medium containing peptides and various postmetabolites. This retention of adenocarcinoma cells in a pre-synthetic period is advantageous because it would provide an opportunity to restore the euploid cell population by suppressing the division of aneuploid cells. A similar possibility was reported by other authors, who studied the influence of different probiotics on cervical or intestinal epithelium [31,32]. Like us, other authors have found an increase in the proportion of cells in the G0/G1 period of the cell cycle. Vielfort et al. reported cell cycle arrest and increased cells in G1 accompanied by the up-regulation of p21 [33]. In contrast, we found not only an increase in cells in G0/G1, but also in those undergoing replication. In our opinion, this could be an indication of checkpoint activation and the induction of programmed cell death in a small cell population (about 2%).

The intestinal epithelium is one of the main barriers between the body and the environment. The microvilli, mucosal layer and glycocalyx prevent the direct contact of macromolecules in the intestinal lumen with the apical surface of the epithelium, and tight junctions limit the paracellular transport of small molecules. However, this is not a passive barrier. Tight junctions are dynamic structures influenced by the gut microbiota and molecules secreted by “good” probiotic bacteria [12]. These secreted molecules exert their effects on tight junctions through a number of biochemical metabolic pathways, including the protein kinase C and MAP kinase pathways, and thereby alter the expression of tight junction proteins, including occludin, ZO-1, ZO-2 and claudins 1, 2, 3 and 4 [34,35]. The structure and strength of tight junctions determine the integrity of the intestinal epithelial monolayer. The main method for determining violations in its integrity is the measurement of transepithelial resistance. Our results showed a strengthening of the monolayer of cultured intestinal epithelial cells in the presence of postmetabolites of Lf 53, which is consistent with data from other groups. *Lactobacillus plantarum* MF1298 and *Ligilactobacillus salivarius* DC5 strains, which have probiotic potential, have been shown to improve epithelial barrier function by increasing the transepithelial resistance of the Caco-2 cell monolayer. The effect was dose- and time-dependent, and this was associated with the increased expression of the ZO-1 protein [13]. In addition, *Lactobacillus casei* was found to restore TER values, cell monolayer permeability and ZO-1 expression caused by the stimulation of Caco-2 cells with TNF-α and IFN-γ. It also stimulates the expression of TLR-2 (Toll-like receptor 2) and p-Akt, which in turn help maintain the integrity of tight junctions in intestinal epithelial cells [14,36].

Like other types of probiotic bacteria, *Limosilactobacillus fermentum* 53 could also protect and help restore the intestinal epithelial barrier. It has been shown that the combination of *Lactobacillus fermentum* L930BB and *Bifidobacterium animalis* subsp. *animalis* IM386, isolated from human intestinal mucosa, triggers specific signaling pathways that lead to actin reorganization and increased cell proliferative potential, thereby helping to maintain the integrity of the intestinal epithelial barrier. These bacteria were found to increase the expression of TLR2 and some growth factors, thereby triggering various signaling cascades in the cell, including the PI3K/Akt and MAPK pathways. The exact way the cascades are triggered has not been established, but it is clear that the combination of the two probiotic strains has application as a potential treatment for chronic inflammatory bowel disease [37].

At least part of the effects we observed on the intestinal epithelial cells could possibly be ascribed to the indole released by *lactobacilli*. It is secreted by tryptophan-processing probiotic bacteria and serves as a signaling molecule for symbionts in the natural microbiota of the gastrointestinal tract. Indole also inhibits the attachment of pathogenic microorganisms to epithelial cells by increasing the expression of genes for the construction of the actin cytoskeleton, for the formation of intercellular contacts and for the secretion of mucin [38].

## 5. Conclusions

Our data on the effects of postmetabolites secreted by *L. fermentum* 53 complement those available in the literature regarding their influence on the proliferative characteristics and barrier functions of HT-29 cells. We found the stimulation of cell division was accompanied by an increase in cell population. This, together with the positive effect on transepithelial resistance, is an indication of the strengthening and stabilization of the cell monolayer, a mandatory condition for the correct performance of barrier functions by enterocytes. Future studies will reveal which of the components secreted by *lactobacilli* are responsible for the observed effects, and the exact cellular and molecular mechanisms underlying them.

## Data Availability

The presented results are part of a research project whose report to the funding institution (Ministry of Education) is in Bulgarian. Primary data are available to interested parties uppon personal communication with the authors.

## References

[1] Culligan E.P., Hill C., Sleator R.D. (2009). Probiotics and gastrointestinal disease: Successes, problems and future prospects. Gut Pathog..

[2] Lee J.E., Lee J., Kim J.H., Cho N., Lee S.H., Park S.B., Koh B., Kang D., Kim S., Yoo H.M. (2019). Characterization of the Anti-Cancer Activity of the Probiotic Bacterium Lactobacillus fermentum Using 2D vs. 3D Culture in Colorectal Cancer Cells. Biomolecules.

[3] Westfall S., Lomis N., Prakash S. (2019). Ferulic Acid Produced by Lactobacillus fermentum Influences Developmental Growth Through a dTOR-Mediated Mechanism. Mol. Biotechnol..

[4] Resta S.C. (2009). Effects of probiotics and commensals on intestinal epithelial physiology: Implications for nutrient handling. J. Physiol..

[5] Lacerda D.C., Trindade da Costa P.C., Pontes P.B., Carneiro Dos Santos L.A., Cruz Neto J.P.R., Silva Luis C.C., de Sousa Brito V.P., de Brito Alves J.L. (2022). Potential role of Limosilactobacillus fermentum as a probiotic with anti-diabetic properties: A review. World J. Diabetes.

[6] Green M., Arora K., Prakash S. (2020). Microbial Medicine: Prebiotic and Probiotic Functional Foods to Target Obesity and Metabolic Syndrome. Int. J. Mol. Sci..

[7] Kaunang T.M.D., Setiawan A.A., Mayulu N., Leonita I., Wijaya A., Yusuf V.M., Mahira M., Yudisthira D., Gunawan W.B., Taslim N.A. (2023). Are probiotics beneficial for obese patients with major depressive disorder? Opinion for future implications and strategies. Front. Nutr..

[8] Sheykhsaran E., Abbasi A., Ebrahimzadeh Leylabadlo H., Sadeghi J., Mehri S., Naeimi Mazraeh F., Feizi H., Bannazadeh Baghi H. (2023). Gut microbiota and obesity: An overview of microbiota to microbial-based therapies. Postgrad. Med. J..

[9] Martin F.P., Wang Y., Sprenger N., Yap I.K., Lundstedt T., Lek P., Rezzi S., Ramadan Z., van Bladeren P., Fay L.B. (2008). Probiotic modulation of symbiotic gut microbial-host metabolic interactions in a humanized microbiome mouse model. Mol. Syst. Biol..

[10] Kerry R.G., Patra J.K., Gouda S., Park Y., Shin H.S., Das G. (2018). Benefaction of probiotics for human health: A review. J. Food Drug Anal.

[11] Kataria J., Li N., Wynn J.L., Neu J. (2009). Probiotic microbes: Do they need to be alive to be beneficial?. Nutr. Rev..

[12] Snoeck V., Goddeeris B., Cox E. (2005). The role of enterocytes in the intestinal barrier function and antigen uptake. Microbes Infect..

[13] Klingberg T.D., Pedersen M.H., Cencic A., Budde B.B. (2005). Application of measurements of transepithelial electrical resistance of intestinal epithelial cell monolayers to evaluate probiotic activity. Appl. Environ. Microbiol..

[14] Eun C.S., Kim Y.S., Han D.S., Choi J.H., Lee A.R., Park Y.K. (2011). Lactobacillus casei prevents impaired barrier function in intestinal epithelial cells. APMIS.

[15] Jariwala R., Mandal H., Bagchi T. (2017). Indigenous lactobacilli strains of food and human sources reverse enteropathogenic E. coli O26:H11-induced damage in intestinal epithelial cell lines: Effect on redistribution of tight junction proteins. Microbiology (Reading).

[16] Ng S.C., Hart A.L., Kamm M.A., Stagg A.J., Knight S.C. (2009). Mechanisms of action of probiotics: Recent advances. Inflamm. Bowel. Dis..

[17] Messaoudi S., Madi A., Prevost H., Feuilloley M., Manai M., Dousset X., Connil N. (2012). In vitro evaluation of the probiotic potential of Lactobacillus salivarius SMXD51. Anaerobe.

[18] O’Hara A.M., Shanahan F. (2007). Mechanisms of action of probiotics in intestinal diseases. ScientificWorldJournal.

[19] <monospace>Petrova M., Georgieva R., Dojchinovska L., Kirilov N., Iliev I., Antonova S., Hadjieva N., Vanova I., Danova S. (2009). Lactic acid bacteria against pathogenic microbes. Trakia J. Sci..

[20] Smith P.K., Krohn R.I., Hermanson G.T., Mallia A.K., Gartner F.H., Provenzano M.D., Fujimoto E.K., Goeke N.M., Olson B.J., Klenk D.C. (1985). Measurement of protein using bicinchoninic acid. Anal Biochem..

[21] Stephanova E., Topouzova-Hristova T., Hazarosova R., Moskova V. (2008). Halothane-induced alterations in cellular structure and proliferation of A549 cells. Tissue Cell.

[22] Commane D.M., Shortt C.T., Silvi S., Cresci A., Hughes R.M., Rowland I.R. (2005). Effects of fermentation products of pro- and prebiotics on trans-epithelial electrical resistance in an in vitro model of the colon. Nutr. Cancer.

[23] Srinivasan B., Kolli A.R., Esch M.B., Abaci H.E., Shuler M.L., Hickman J.J. (2015). TEER measurement techniques for in vitro barrier model systems. J. Lab. Autom..

[24] Jochems P.G.M., Garssen J., van Keulen A.M., Masereeuw R., Jeurink P.V. (2018). Evaluating Human Intestinal Cell Lines for Studying Dietary Protein Absorption. Nutrients.

[25] Ponce de Leon-Rodriguez M.D.C., Guyot J.P., Laurent-Babot C. (2019). Intestinal in vitro cell culture models and their potential to study the effect of food components on intestinal inflammation. Crit. Rev. Food Sci. Nutr..

[26] Fois C.A.M., Le T.Y.L., Schindeler A., Naficy S., McClure D.D., Read M.N., Valtchev P., Khademhosseini A., Dehghani F. (2019). Models of the Gut for Analyzing the Impact of Food and Drugs. Adv. Healthc. Mater..

[27] Scheppach W., Bartram P., Richter A., Richter F., Liepold H., Dusel G., Hofstetter G., Ruthlein J., Kasper H. (1992). Effect of short-chain fatty acids on the human colonic mucosa in vitro. JPEN J. Parenter. Enteral. Nutr..

[28] Kang S.J., Kim M.J., Son D.Y., Kang S.S., Hong K.W. (2022). Effects of Spore-Displayed p75 Protein from Lacticaseibacillus rhamnosus GG on the Transcriptional Response of HT-29 Cells. Microorganisms.

[29] Wu H., Xie S., Miao J., Li Y., Wang Z., Wang M., Yu Q. (2020). Lactobacillus reuteri maintains intestinal epithelial regeneration and repairs damaged intestinal mucosa. Gut Microbes.

[30] Zhang Y., Alexander P.B., Wang X.F. (2017). TGF-beta Family Signaling in the Control of Cell Proliferation and Survival. Cold Spring Harb. Perspect. Biol..

[31] Dehghani N., Tafvizi F., Jafari P. (2021). Cell cycle arrest and anti-cancer potential of probiotic Lactobacillus rhamnosus against HT-29 cancer cells. Bioimpacts.

[32] Abedi A., Tafvizi F., Jafari P., Akbari N. (2024). The inhibition effects of Lentilactobacillus buchneri-derived membrane vesicles on AGS and HT-29 cancer cells by inducing cell apoptosis. Sci. Rep..

[33] Vielfort K., Weyler L., Soderholm N., Engelbrecht M., Lofmark S., Aro H. (2013). Lactobacillus decelerates cervical epithelial cell cycle progression. PLoS One.

[34] Ramakrishna B.S. (2009). Probiotic-induced changes in the intestinal epithelium: Implications in gastrointestinal disease. Trop Gastroenterol..

[35] Zyrek A.A., Cichon C., Helms S., Enders C., Sonnenborn U., Schmidt M.A. (2007). Molecular mechanisms underlying the probiotic effects of Escherichia coli Nissle 1917 involve ZO-2 and PKCzeta redistribution resulting in tight junction and epithelial barrier repair. Cell Microbiol..

[36] Cario E., Gerken G., Podolsky D.K. (2007). Toll-like receptor 2 controls mucosal inflammation by regulating epithelial barrier function. Gastroenterology.

[37] Paveljsek D., Juvan P., Kosir R., Rozman D., Hacin B., Ivicak-Kocjan K., Rogelj I. (2018). Lactobacillus fermentum L930BB and Bifidobacterium animalis subsp. animalis IM386 initiate signalling pathways involved in intestinal epithelial barrier protection. Benef. Microbes.

[38] Bansal T., Alaniz R.C., Wood T.K., Jayaraman A. (2010). The bacterial signal indole increases epithelial-cell tight-junction resistance and attenuates indicators of inflammation. Proc. Natl. Acad. Sci. USA.

